# Scoparone Inhibits LPS-Simulated Inflammatory Response by Suppressing IRF3 and ERK in BV-2 Microglial Cells

**DOI:** 10.3390/molecules21121718

**Published:** 2016-12-14

**Authors:** Duk-Yeon Cho, Hyun Myung Ko, Joonsoo Kim, Byung-Wook Kim, Yo-Sep Yun, Jeong-In Park, Palanivel Ganesan, Jin-Tae Lee, Dong-Kug Choi

**Affiliations:** 1 Department of Biotechnology, College of Biomedical and Health Science, Konkuk University, Chungju 380-701, Korea; ejrdus1026@naver.com (D.-Y.C.); 997176@kku.ac.kr (H.M.K.); kgfdkr@gmail.com (J.K.); Kim.Byungwook@mayo.edu (B.-W.K.); aaa003132@naver.com (Y.-S.Y.); pji0819@hanmail.net (J.-I.P.); palanivel@kku.ac.kr (P.G.); 2 Nanotechnology Research Center and Department of Applied Life Science, College of Biomedical and Health Science, Konkuk University, Chungju 380-701, Korea; 3 Department of Cosmeceutical Science, Daegu Haany University, Gyungsan 38610, Korea; jtlee@dhu.ac.kr

**Keywords:** scoparone, IRF-3, ERK, microglial cells

## Abstract

Microglia activation and the release of various inflammatory cytokines are largely related to neurological diseases, including Parkinson’s, Alzheimer’s, and other brain diseases. The suppression of microglial cells using natural bioactive compounds has become increasingly important for brain therapy owing to the expected beneficial effect of lower toxicity. Scoparone (6,7-dimethoxycoumarin), a major bioactive compound found in various plant parts, including the inner shell of chestnut (*Castanea crenata*), was evaluated on lipopolysaccharide (LPS)-activated BV-2 microglia cells. The results indicated that scoparone suppresses the LPS-stimulated increase of neuroinflammatory responses and inhibited the pro-inflammatory cytokine production in the BV-2 microglial cells. A mechanistic study showed that scoparone specifically inhibited the LPS-stimulated activation via a major regulation of IRF-3 and a regulation of ERK, whereby the phosphorylation in the BV-2 microglial cells is blocked. These data suggest that scoparone has anti-neuroinflammatory effects in LPS-activated BV-2 microglial cells, and could possibly be used in the development of novel drugs for the prevention and treatment of neuroinflammatory diseases.

## 1. Introduction

Neurons and microglial cells are the integral part of the central nervous system, in which the microglial cells occupy 5%–10% of the brain cells [1,2,3]. The microglial cells in the brain control various protective mechanisms of brain cells, and during uncontrolled conditions they also produce various mediators, such as inducible nitric-oxide synthase (iNOS) and cyclooxygenase-2 (COX-2); cytokines like tumor-necrosis factor-α (TNF-α), interleukin-1β (IL-1β), and interleukin-6 (IL-6); and chemokines that lead to neuronal damage and brain-cell death [4]. It has also been reported that the progression of neurodegenerative diseases can be reduced with the suppression of microglial cell activation [4,5,6].

Toll-like receptors (TLRs) recognize the pathogen-associated molecular patterns (PAMPs) and the damage-associated molecular patterns (DAMPs), and TLR4 has also recognized LPS [7]. TLR4 transduces the phosphorylation of the two adaptor proteins TIR-domain-containing adapter-inducing interferon-β (TRIF) and myeloid differentiation primary response gene 88 (MyD88) downstream. LPS-stimulated TRIF-dependent signaling induces the transcription factor, interferon regulatory factor 3 (IRF3); otherwise, MyD88-dependent signaling transduces another type of transcription factor, NF-κB [8]. Also, IRF3 plays an important role in the innate immune system’s response to viral infection. IRF-3 is LPS-stimulated TLR4-TIRF dependent activated after phosphorylation, facilitating its dimerization and interaction of coactivators such as CBP and p300. The activated IRF-3 complex then translocate to the nucleus where it regulates the transcription of target genes via IFN-β and iNOS [8,9,10].

Scoparone (6,7-dimethoxycoumarin) is one of the major active natural bioactive compounds in chestnut inner shell (*Castanea crenata*), which has multiple beneficial activities including anticancer, anti-coagulant, antitumor, and anti-inflammatory properties [11,12,13,14]. Scoparone has a very high potential regarding a free-radical scavenging activity that resulted in the lowering of the plasma lipids in an alloxan-fed diabetic rabbit [15]. Further, scoparone has the neurite-outgrowth potential through its stimulation of the upstream steps of ERK, cyclic ACP-dependent protein kinase, protein-kinase C, and CA^2+^/calmodulin kinase II, and it has reduced the cytotoxicity induced by l-DOPA [16,17]. Some researchers also reported that scoparone has protective effects against acute lung injury and human umbilical-vein endothelial cells [18,19]. The protective role of scoparone against neuroinflammation in the microglial cells has not been reported, therefore, the focus of the present study is the evaluation of scoparone’s anti-neuroinflammatory effects and its molecular mechanism in LPS-induced BV-2 microglial cells.

## 2. Results

### 2.1. Effect of Scoparone on LPS-Induced Cellular Viability and Nitrite Production in BV-2 Microglial Cells

**Figure 1:**
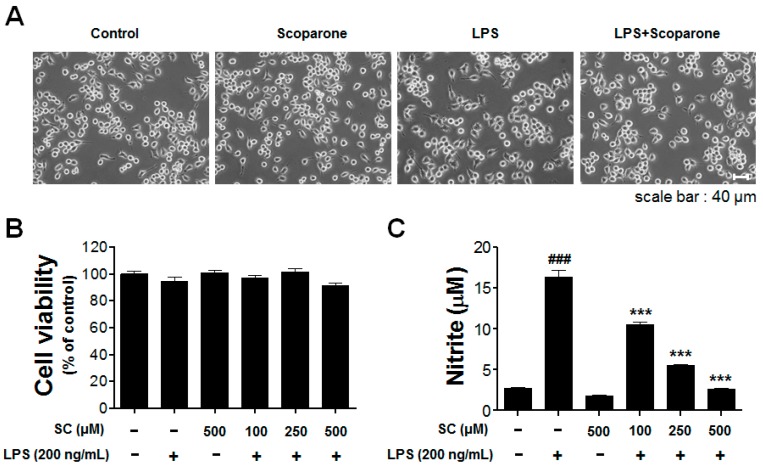
Effect of scoparone on cell viability and nitric-oxide production in LPS-induced BV-2 microglial cells. The morphological changes are represented in the BV2 microglial cells (**A**). The viability in the scoparone-treatment cells was evaluated using the MTT assay (**B**). The BV-2 microglial cells were incubated with 100 μM, 250 μM, and 500 μM of scoparone for 24 h. The results are displayed as a percentage of the control samples. The nitrite in the medium was determined using the Griess assay (**C**). Data are the mean ± standard error (*n* = 3) of three independent experiments. ### *p* < 0.005, compared with the control group; *** *p* < 0.005 compared with the LPS-treated group.

The authors aimed to evaluate the effect of scoparone on the NO production in LPS-stimulated BV-2 microglial cells. In addition, we examined the cell morphology of the BV2 microglial cells that were incubated with scoparone (500 μM) in the presence or absence of LPS (200 ng/mL). The shape of the LPS-treated microglial cells was ramified compared to the control group, indicating activation of the microglial cells. This morphological change induced by LPS treatment was successfully inhibited by pretreatment with scoparone (Figure 1A).

BV-2 microglial cells were initially treated with various concentrations of scoparone (100 μM, 250 μM, and 500 μM) and/or LPS (200 ng/mL) to determine whether they exert any toxicity onto the cells, and this was determined using a 3-(4,5-dimethylthiazol-2-yl)-2,5-diphenyltetrazolium bromide (MTT) assay. A pretreatment of scoparone at concentrations of 100 μM, 250 μM, and 500 μM with LPS (200 ng/mL) did not affect the cell viability in the BV-2 microglial cells (Figure 1B). To evaluate the effect of scoparone on LPS-induced NO production, the BV-2 microglial cells were pretreated for 30 min with different concentrations of scoparone (100 μM, 250 μM, and 500 μM), followed by the LPS (200 ng/mL) treatment for 24 h, and the levels of NO in the culture media were determined using the Griess assay. The LPS alone markedly increased the NO production (16.4 ± 1.5 μM) compared with that of the control (2.8 ± 0.3 μM). Scoparone alone does not change the NO level from the comparison with the control group. A significant suppression of the levels of the NO production in a concentration-dependent manner to 10.49 ± 0.7 μM, 5.51 ± 0.3 μM, and 2.60 ± 0.4 μM, respectively, was observed in the LPS-stimulated BV-2 cells (Figure 1C).

### 2.2. Effect of Scoparone on Levels of iNOS-mRNA Expression and Protein Induction in BV-2 Microglial Cells

**Figure 2:**
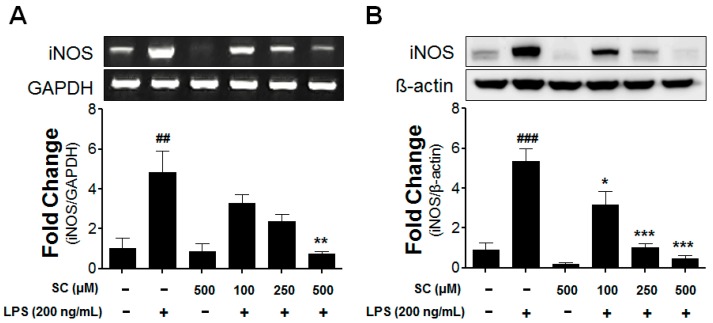
Effect of scoparone on LPS-induced iNOS-mRNA and iNOS-protein expressions in BV-2 microglial cells. The BV-2 microglial cells were seeded at 2.5 × 10^5^ cells/mL, and were incubated for 6 h and 20 h with various concentrations of scoparone 1 h before the stimulation with LPS. The mRNA was first isolated, and the mRNA expression was then evaluated using the RT-PCR (**A**). The cell lysates were electrophoresed, and the iNOS expressions were detected using a specific antibody (**B**). The data are the mean ± standard error (*n* = 3) of three independent experiments. ## *p* < 0.01, ### *p* < 0.005, compared with the control group; * *p* < 0.05, ** *p* < 0.01, and *** *p* < 0.005 compared with the LPS-treated group.

Since the scoparone treatment reduced the NO concentration, the mRNA and protein levels of the iNOS were evaluated for a further investigation. As shown in Figure 2A, LPS stimulation (200 ng/mL) appreciably upregulated the expression of the iNOS-mRNA level to 4.83 ± 1.9 folds after 6 h in the comparison with the control group. The scoparone treatment inhibited the iNOS mRNA in the LPS-stimulated BV-2 microglial cells in a concentration-dependent manner. The protein levels of the iNOS were also assessed using a western blot analysis. At 20 h after the LPS stimulation, the iNOS-protein level was markedly elevated to 5.34 ± 1.6 folds in the comparison with the control group (Figure 2B). These data correlated well with the reduction of the corresponding mRNA levels, suggesting that scoparone is a potential anti-inflammatory agent owing to the reduction of the iNOS expression in LPS-induced BV-2 microglia cells.

### 2.3. Effect of Scoparone on COX-2-mRNA Expression and Protein Induction on LPS-Induced BV-2 Microglial Cells

**Figure 3:**
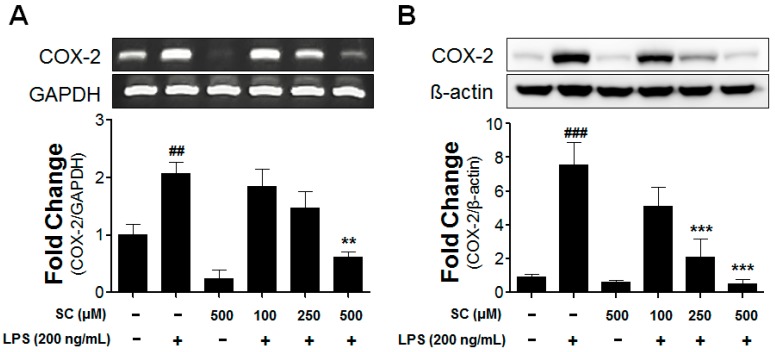
Effect of scoparone on LPS-induced COX-2-mRNA and COX-2-protein expressions in BV-2 microglial cells. The BV-2 microglial cells were seeded at 2.5 × 10^5^ cells/mL, and were incubated for 6 h and 20 h with various concentrations of scoparone 1 h before the stimulation with LPS. The mRNA was first isolated, and the mRNA expression was then evaluated using the RT-PCR (**A**). The cell lysates were electrophoresed, and the COX-2 expressions were detected using a specific antibody (**B**). The data are the mean ± standard error (*n* = 3) of three independent experiments. ## *p* < 0.01, ### *p* < 0.005, compared with the control group; ** *p* < 0.01 and *** *p* < 0.005 compared with the LPS-treated group.

Prostaglandins (PGEs) are crucial factors in the event of neuroinflammation in the brain. The synthesizing enzyme COX-2 is one of the most-referred pro-inflammatory mediators along with iNOS. To evaluate the expression of the COX-2 mRNA and protein in the BV-2 microglial cells, the enzyme was treated with LPS in the absence or presence of scoparone for 6 h or 20 h. Aspects of the expression of the COX-2 mRNA were changed after 6 h according to an RT-PCR analysis. The stimulation with LPS significantly increased the COX-2 gene expression compared with that in the control group; however, a pretreatment with scoparone before the LPS stimulation for 6 h inhibited the COX-2-mRNA expression in the LPS-stimulated BV-2 microglial cells in a dose-dependent manner (Figure 3A). Moreover, the COX-2-protein level in the LPS-stimulated BV-2 microglial cells was dramatically reduced dose-dependently by the scoparone treatment (Figure 3B).

### 2.4. Inhibitory Effect of Scoparone on Proinflammatory-Cytokine Expression in BV-2 Microglial Cells

**Figure 4:**
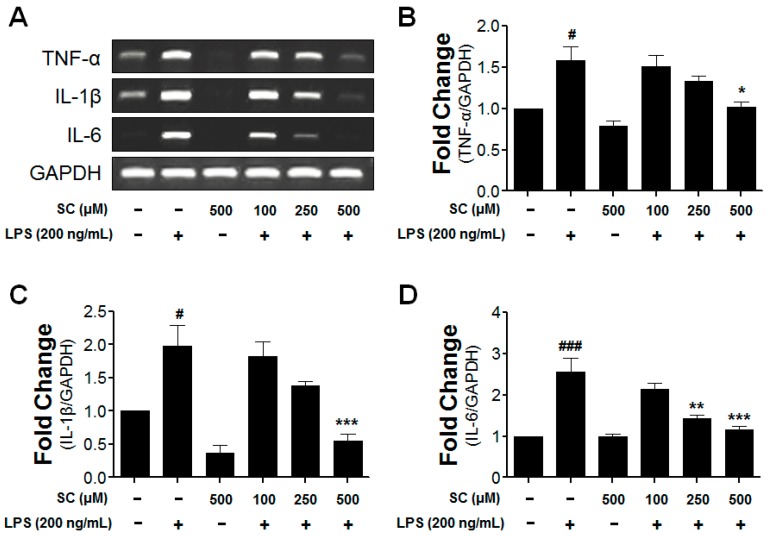
Effect of scoparone on mRNA expression of LPS-induced cytokines in BV-2 microglial cells. The BV-2 microglial cells were seeded at 2.5 × 10^5^ cells/mL, and were incubated for 6 h with various concentrations of scoparone 1 h before the stimulation with LPS. The mRNA was first isolated, and the mRNA expressions of the tumor-necrosis factor (TNF)-α, interleukin (IL)-1β, and interleukin (IL)-6 were then evaluated using the RT-PCR (**A**). The representative densitometry analyses of TNF-α (**B**), IL-1β (**C**) and IL-6 (**D**) compared with GAPDH mRNA. The data are the mean ± standard error (*n* = 3) of three independent experiments. # *p* < 0.05, ### *p* < 0.005, compared with the control group; * *p* < 0.05, ** *p* < 0.01, and *** *p* < 0.005 compared with the LPS-treated group.

Previous reports suggest that the expression of proinflammatory cytokines such as TNF-α, IL-1β, and IL-6 from BV-2 microglial cells is an indicator of neuronal-cell damage [20]; therefore, the possibility of the inhibitory effect of scoparone on the LPS-induced BV-2 microglial cells was investigated. The RT-PCR analysis was used to examine whether the suppression of TNF-α, IL-1β, and IL-6 by scoparone is due to a decrease of the mRNA in the BV-2 microglial cells. While TNF-α,IL-1β, and IL-6 were not expressed at detectable levels under the normal culture conditions, the expressions of these cytokines were significantly up-regulated after 6 h of treatment with LPS (200 ng/mL). The LPS-stimulated mRNA levels of the proinflammatory cytokines (TNF-α, IL-1β, and IL-6) were reduced by the scoparone treatment (Figure 4), suggesting that the scoparone negatively regulated the production of TNF-α, IL-1β, and IL-6 at the transcriptional level in the LPS-induced BV-2 microglial cells. The rep-resentative quantification data revealed that LPS-stimulated proinflammatory cytokine mRNA levels (TNF-α, IL-1β and IL-6) were significantly decreased following scoparone treatment (Figure 4B–D).

### 2.5. Effect of Scoparone on LPS-Induced Phosphorylation of IRF3 and ERK in BV-2 Microglial Cells

**Figure 5:**
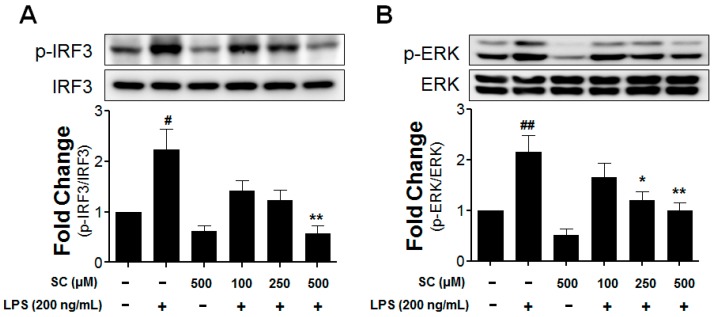
Effect of scoparone on LPS-induced IRF3 and ERK activations induced by LPS in BV-2 microglial cells. The BV-2 microglial cells were seeded at 2.5 × 10^5^ cells/mL, and were incubated for 1 h with various concentrations of scoparone 1 h before the stimulation with LPS. The cell lysates were electrophoresed, and the phospho-IRF3 (**A**), phospho-ERK (**B**), and ERK expressions were detected using a specific antibody. # *p* < 0.05, ## *p* < 0.01, compared with the control group; * *p* < 0.05, ** *p* < 0.01 compared with the LPS-treated group.

The authors investigated the main LPS-induced TLR4 downstream-signaling pathways to determine the way that scoparone inhibits the production of pro-inflammatory mediators and cytokines. Following the LPS treatment of the BV-2 cells, various defensive responses are activated including IRF3. As is known with TRIF-dependent signaling, the IRF3 regulates the expression of iNOS and other cytokines [21]. The effects of scoparone pretreatment on the LPS-induced phosphorylation of IRF3 and ERK in BV-2 microglial cells are shown in Figure 5. Compared with the control in the LPS-induced BV-2 microglial cells, after 1 h, the phosphorylation of the IRF3 upregulated it to 2.23 ± 0.7 fold, and the ERK was upregulated to 2.16 ± 0.7 fold. The LPS-stimulated BV-2 cells were pretreated with scoparone (100 μM, 250 μM, and 500 μM). The IRF3 and ERK phosphorylation were inhibited in a concentration-dependent manner in the LPS-induced BV-2 microglial cells. 

**Figure 6:**
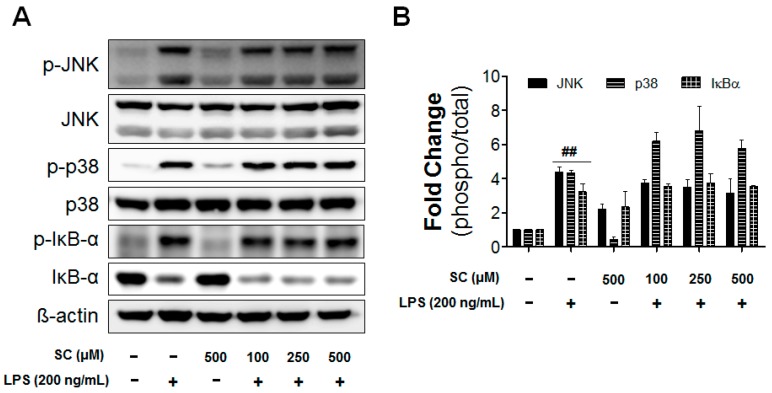
Effect of scoparone on LPS-induced MAPKs and NF-κB activation in BV-2 microglial cells. The BV-2 microglial cells were seeded at 2.5 × 10^5^ cells/mL, and were incubated for 1 h with various concentrations of scoparone 1 h before the stimulation with LPS. The cell lysates were electrophoresed, and the phospho-JNK, JNK, phospho-p38, p38 MAPKs, phospho-IκB-α, and IκB-α expressions were detected using a specific antibody (**A**). The quantification data are shown in the right panel (**B**). Data are the mean ± standard error (*n* = 3) of three independent experiments. ## *p* < 0.01, compared with the control group.

On the other hand, NFκB, p38, and JNK are among the most important molecules in the signaling pathways that control the synthesis and release of proinflammatory substances by activated microglia [22].The possibility was raised that LPS induced proinflammatory mediators by MAPKs. However, scoparone treatments didn’t show any significant effects on ERK, p38, and JNK (Figure 6). Taken together, the results showed that scoparone significantly inhibited the phosphorylation of IRF-3 and ERK, but not NFκB, p38, and JNK by LPS in BV-2 microglial cells.

## 3. Discussion

The activation of the microglia resulted in the elevated levels of certain neurotoxic mediators and proinflammatory mediators, which can result in the severe damage of brain cells, and in turn lead to various neuroinflammatory diseases [4,23]. It is crucial to prevent the activation of the microglial cells so that various neuroinflammatory diseases can also be prevented. Several studies show that the activation of the microglial cells can be effectively prevented by bioactive compounds that have been isolated from natural resources [4,5,24].

As mentioned previously, scoparone is very effective in various bioactivities, including antioxidant, anticoagulant, and anticancer effects, through its downregulation of various proinflammatory cytokines [14,25,26]. This study is focused on the effective role of scoparone in the prevention of the neuroinflammations in LPS-mediated BV-2 microglial cells. The current study confirmed that scoparone can inhibit the activation of LPS-activated microglial cells, thereby indicating that it might efficiently prevent various neuroinflammatory diseases. Several other studies also showed that the bioactive compounds of plants can efficiently reduce the production of nitrite [27]. Controlling the release of NO and PGE2 would efficiently inhibit the excessive inflammation that causes chronic diseases, including neurodegenerative diseases [28]. The authors also studied the scoparone treatment in terms of an inhibitory activation of inflammatory mediators and proinflammatory cytokines such as TNF-α, IL-1β, and IL-6 at various molecular levels. This study confirmed that various treatments of scoparone showed inhibited inflammatory mediators in BV-2 microglial cells. According to the study of Jang et al., similar results regarding the anti-inflammatory action of scoparone were shown [29].

In the present study, scoparone significantly inhibited both IRF3 and ERK activation in LPS-stimulated BV-2 microglial cells. It is possible that scoparone inhibits LPS-induced TRIF and MyD88 activation via the TLR4 signaling pathway. In TLR4, or other TLRs, IRF3 is activated by a TRIF-dependent signal, and it is a well-known transcriptional factor that has been reported in a number of papers [30]. Specifically, in a knock-out mouse-model experiment conducted by Petrasek et al., the reduction of the IRF3-signal transduction showed a protective effect in an alcohol-induced liver-tissue lesion [31]. MAPKs are also involved in the production of the LPS-induced COX-2 and iNOS in microglial cells. One of the important MAPK families, ERK, is positively related to the LPS signaling in microglial cells, and it responds to proinflammatory cytokines [32,33]. NFκB, p38MAPK, and JNK have especially been implicated in the signal-transduction pathways that are responsible for increased iNOS-, COX-2-, TNF-α-, IL-1β-, and IL-6-gene expressions in glial cells [34]. The present study revealed that scoparone did not affect the activation of both NFκB and MAPKs such as p38 and JNK that are induced in BV-2 microglial cells by LPS stimulation (Figure 6). The results of the present study show that scoparone mainly decreased the activation of the IRF3 level, indicating that scoparone could alleviate neuroinflammation by reducing the TRIF-dependent signaling molecule, IFR3, in LPS-stimulated microglial cells.

## 4. Materials and Methods

### 4.1. Materials

Scoparone (6,7-dimethoxycoumarin), lipopolysaccharide (*Escherichia coli*; 055:B5), 3-(4,5-dimethylthiazol-2-yl)-2,5-diphenyltetrazolium bromide (MTT), *N*-(1-naphthyl)ethylenediamine dihydrochloride, sulfanilamide, dimethyl sulfoxide (DMSO), and sodium nitrite were obtained from Sigma-Aldrich (St. Louis, MO, USA). The phosphate-buffered saline, Dulbecco’s modified Eagle medium (DMEM), and fetal bovine serum (FBS) were obtained from Gibco/Invitrogen (Carlsbad, CA, USA). The inhibitors for protease and phosphatase were obtained from Roche (Indianapolis, IN, USA). The tissue-culture plate and the 100 mm culture dishes were obtained from Nunc Inc. (Aurora, IL, USA). The iNOS antibody was obtained from Calbiochem (La Jolla, CA, USA).

### 4.2. BV-2 Microglial-Cell Culture

The BV2 microglia cells were acquired as described previously [35,36]. The BV-2 microglial cells were cultured in DMEM supplemented with 5% FBS and 1% of 100 units/mL of penicillin/streptomycin at 37 °C in a humidified 5%-CO_2_ incubator. In all of the experiments, the cells were seeded at a density of 2.5 × 10^5^ cells/mL, and they were 6 h after then pretreated for 1 h with different concentrations of scoparone (100 μM, 250 μM, and 500 μM), followed by an incubation with LPS (200 ng/mL) for the indicated times (1 h, 6 h, and 24 h). In the cell morphology experiment, the BV2 microglial cells were pretreated with 500 μM of scoparone followed by LPS (200 ng/mL) induction for 24 h, and the cellular morphology images were observed by using phase contrast microscopy (Axio; Carl Zeiss, Jena, Germany).

### 4.3. Cell Viability and Nitrite Assay

The BV-2 microglial cells that were seeded at a density of 2.5 × 10^5^ cells/mL were pretreated with various concentrations of scoparone (100 μM, 250 μM, and 500 μM) for 1 h, followed by the LPS (200 ng/mL) for 24 h. The viability of cells were measured next according to the modified method of Kim et al., (2015) [5]. For the nitrite assay, the BV-2 microglial cells that were seeded at a density of 2.5 × 10^5^ cells/mL were pretreated with various concentrations of scoparone (100 μM, 250 μM, and 500 μM) for 1 h, followed by the LPS (200 ng/mL) for 24 h. A further procedure of the nitrite assay was followed according to the modified method of Kim et al. [4]. All of the experiments were repeated for three individual sets.

### 4.4. Total RNA Isolation and Reverse Transcription Polymerase Chain Reaction (RT-PCR)

The total RNA was extracted from the BV-2 microglial cells using a Trizol reagent (Invitrogen Life Technologies, Carlsbad, CA, USA) according to the manufacturer’s instructions. A first-strand cDNA synthesis was performed using 2.5 μg of total RNA and the ReverTra Ace-α kit (Toyobo, Osaka, Japan). The reaction was performed at 60 °C for 60 min and was heated at 95 °C for 5 min; 1 μL from each RT-reaction mixture was used for a PCR amplification. The PCR amplification was performed using specific primers (Bioneer, Daejeon, Korea), as reported previously [5]. All of the PCR products were resolved using 1.2% agarose-gel electrophoresis and were visualized with ethidium bromide. For quantification, the gels were photographed, the pixel intensity for each band was determined in Image J (NIH), and they were normalized to the band intensity of GAPDH mRNA.

### 4.5. Western Blot Analysis

The cells were washed twice with PBS, placed at 4 °C, and lysed for 10 min in lysis buffer (1× RIPA lysis buffer, protease-inhibitor cocktail, and phosphatase-inhibitor cocktail). The lysates were centrifuged at 14,000 rpm and 4 °C, and the supernatants were collected for further analysis. Equal amounts of protein (20 μg or 40 μg) were separated electrophoretically using a 10% sodium dodecyl sulfate-polyacrylamide electrophoresis, and the resolved proteins were transferred to polyvinylidene-difluoride membranes (Millipore, Bedford, MA, USA). The membranes were incubated for 1 h with 5% skim milk in the PBS buffer to block the nonspecific binding. The membranes were then incubated with primary antibodies to anti-iNOS, anti-phospho ERK, and anti-phospho IRF3 (1:2000; Cell Signaling Technology, Danvers, MA, USA), anti-COX-2 (1:2000; Santa Cruz Biotechnology, Santa Cruz, CA, USA), and anti-β-actin (1:2000; Cell Signaling Technology, Danvers, MA, USA). The blots were visualized with an enhanced chemiluminescence detection system (Santa Cruz Biotechnology) according to the recommended procedure.

### 4.6. Statistical Analysis

All of the data were analyzed using the Graph Pad Prism ver. 5.01 (Graph Pad, Inc., La Jolla, CA, USA). All of the data are expressed as the mean ± standard error of at least three independent experiments that were performed in triplicate. The statistical analysis was performed with a one-way analysis of variance followed by Tukey’s multiple-comparison test. *p*-Values < 0.05 were considered statistically significant.

## 5. Conclusions

From the above results, it was confirmed that scoparone, a compound isolated from the inner shell of *Castabea crebata*, exerts anti-neuroinflammatory effects in LPS-activated BV-2 microglial cells. This finding represents a great potential regarding the further development of drugs for the treatment of various neuroinflammatory disorders including Parkinson’s Diseases. Further studies should focus on the role of these compounds in vivo with respect to various neurological-disease models.
